# Heterogeneity of Tumors in Breast Cancer: Implications and Prospects for Prognosis and Therapeutics

**DOI:** 10.1155/2020/4736091

**Published:** 2020-10-08

**Authors:** Nigatu Tuasha, Beyene Petros

**Affiliations:** Addis Ababa University, College of Natural Science, Department of Microbial, Cellular and Molecular Biology, P.O. Box 1176, Addis Ababa, Ethiopia

## Abstract

Breast cancer is the most commonly diagnosed form of cancer in women comprising 16% of all female cancers. The disease shows high intertumoral and intratumoral heterogeneity posing diagnostic and therapeutic challenges with unpredictable clinical outcome and response to existing therapy. Mounting evidence is ascertaining that breast cancer stem cells (CSCs) are responsible for tumor initiation, progression, recurrence, evolution, metastasis, and drug resistance. Therapeutics selectively targeting the CSCs based on distinct surface molecular markers and enhanced intracellular activities of these cells continue to evolve and hold significant promise. Having plethora of heterogeneity accompanied with failure of existing conventional therapeutics and poor prognosis, the present review focuses on elucidating the main signaling pathways in breast CSCs as major therapeutic targets. The role of developments in nanomedicine and miRNA as targeted delivery of therapeutic anticancer agents is also highlighted.

## 1. Introduction

Cancer is a group of diseases where cells in the body grow and divide without normal control [1]. It remains a global public health threat despite therapeutic endeavor over the years [2]. Cancer could be grouped as carcinoma (arises from the epithelial cells), sarcoma (tumor of connective or supportive tissues), lymphoma and myeloma (of the cells of the immune system), leukemia (starts in blood forming tissue), and brain and spinal cord cancers (of the central nervous system). To date, there are about 200 known types of cancers with many subtypes and there is no single cause for any one type. Next to cardiovascular diseases, cancer is the second leading cause of global death where about 18.1 million new cancer cases and 9.6 million deaths occurred in 2018 [3–5]. Lung and breast cancer accounted each for 2.09 million new cases in 2018 [3, 6]. Colorectal and prostate cancers followed with 1.8 million and 1.28 million cases, respectively, in the same year [6]. The most common causes of cancer deaths worldwide in 2018 include lung (1.76 million), colorectal (862, 000), stomach (783, 000), liver (782, 000), and breast (627, 000) cancers [6]. Overall, 57 % of new cancer cases, 65 % of the cancer deaths, and 48 % of the 5-year prevalent cancer cases occurred in the less developed regions of the world [4].

Breast cancer remains number one killer among females in less developed countries while it is the second leading cause of cancer death among females in more developed countries, next to lung cancer [4, 7]. Metastasis to the lungs, bone, and the brain are main causes of mortality in breast cancer patients.

The World Health Organization (WHO) estimates that the number of new cases will rise by about 70% over the next 2 decades. The main factors are said to be the growth and aging of the population and an increasing prevalence of established risk factors such as smoking, overweight, physical inactivity, and changing reproductive patterns associated with urbanization and economic development. It is asserted that around one third of deaths from cancer are due to the five leading behavioral and dietary risks: high body mass index, low fruit and vegetable intake, lack of physical activity, tobacco use, and alcoholism [5, 7].

Given the above epidemiological data on breast cancer and the failure of available treatments and poor patient outcomes, identifying the root cause of the problem and finding a novel treatment target remains indispensable. The present review discusses the diversity of breast cancer subtypes and intratumoral heterogeneity and its mechanisms, the biology of breast cancer stem cells (CSCs), their identification markers, and major signaling pathways taking a position that breast CSCs could be used as a potential therapeutic target for a durable breast cancer management approach. Advancements in nanomedicine and the role of miRNA in the direction of beating breast CSCs by a better diagnosis and therapeutics are also discussed.

## 2. Breast Cancer: Epidemiology and Risk Factors

Breast cancer is the most commonly diagnosed cancer of all cancers in women. It comprises 25% both in the developed and less developed world [4, 5]. It comes next to lung cancer as an overall cause of death for women (15.4% in more developed and 14.3% in less developed world) [4, 8]. Around the world, there is no population and woman with a truly low risk of developing breast cancer these days. Since the 2008 estimates by GLOBOCAN (an International Agency for Research on Cancer—IARC), both breast cancer incidence and mortality have increased by more than 20% and 14%, respectively [9]. The incidence rate of breast cancer varies from 19.3 per 100,000 women in Eastern Africa to 89.7 per 100,000 women in Western Europe [10]. Better awareness about the disease, identification of its early signs, and availability of screening programs are contributing to variable incidence rates across different regions of the world [11]. In most of the developing regions, the incidence rates are below 40 per 100,000 [5]. In advanced breast cancer, brain metastases develop in approximately 10–16% of patients and are associated with poor prognosis and survival. Different subtypes of breast cancer are associated with different risks of developing central nervous system metastases, however [12].

Mortality is relatively low in most of the low-incidence countries, but the likelihood that an individual dies of breast cancer is much higher (nearly 17%) in low-incidence countries than in high-incidence countries [13]. The reasons for the differential survival are multiple and include cultural influences, stage of presentation, and standards of healthcare [14]. Among the established risk factors are being a female, early onset of menstruation, late onset of menopause, long menstrual history, use of oral contraceptives, never having children/having them later in life, age, family history, genetics, personal history of breast cancer, radiation to chest or face before age of 30, race/ethnicity, pregnancy, and breastfeeding [2, 13, 15]. Potentially avoidable risk factors include overweight/obesity, using hormone replacement therapy (HRT), drinking alcohol, smoking, and lack of exercise [15, 16]. Low levels of vitamin D [17], light exposure at night [18], certain kinds of noncancerous breast diseases [19], and exposure to multiple sources of polycyclic aromatic hydrocarbons from the environment [20] are among the emerging risk factors for breast cancer. Recent finding reported that women with dense breasts have a roughly 2-fold higher breast cancer risk relative to women with nondense breasts [21]. Height (above average) was positively associated with risk of all breast cancer molecular subtypes [22]. Grilled, barbecued, and smoked meats were reported not only as increasing the risk for breast cancer but also as increasing the death risk for breast cancer survivors [23].

Previously, it was argued that risk of breast cancer is lower among low- and middle-income countries [24]. Lifestyle including nature of food consumed was the main reason for low incidence in those countries [25]. Nowadays, however, this belief is only a myth as around 45% of the cases and 55% of deaths due to breast cancer occur in low- and middle-income countries, according to recent estimates [11, 26]. Adoption of “western lifestyle” is largely implied in increasing the incidence and burden of breast cancer in low- and middle-income countries [11, 27].

Male breast cancer is an uncommon form that comprises less than 1% of all breast cancers globally, although an increasing trend in incidence is seen recently [28]. Due to its rarity, there are few clinical trials, and many clinical recommendations are, hence, derived from studies of female breast cancer [29]. Relatively little is known about the etiology of male breast cancer. Epidemiologic risk factors for male breast cancer encompass disorders related with hormonal imbalances and radiation exposure [29, 30].

## 3. Diversity in Tumors of Breast Cancer and the Mechanisms of Heterogeneity

Human breast cancer is a group of highly heterogeneous lesions of about 20 different subtypes, morphologically [31]. It is highly heterogeneous in terms of its etiology and pathological characteristics, and some show slow growth with excellent prognosis, whereas others are clinically aggressive [32]. Understanding the specific driving forces behind different subtypes of cancer is indispensable for better management of the disease [33]. Besides, development of more effective treatments against breast cancer necessitates thorough understanding of the molecular mechanisms involved in breast tumor development and the acquisition of malignancy [34].

The mechanisms accounting for breast cancer heterogeneity remain elusive [35]. However, two conventional theories (clonal evolution and CSC) hold possible explanations [31]. The clonal evolution theory is the first model to describe a way in which cancer cells with diverse phenotypes could arise within a tumor. It states that distinct cancer cell populations evolve progressively due to heritable genetic and epigenetic changes during a multistep tumorigenesis process [33]. These random events create conducive environment for the selection and clonal outgrowth of novel cell populations resulting from accumulating mutations [36].

The CSC model suggests that cancer cells with similar genetic backgrounds can be hierarchically organized according to their tumorigenic potential [37]. Accordingly, CSCs reside at the apex of the hierarchy and are thought to possess the majority of a cancer's tumor-initiating and metastatic ability [35]. Unidirectional nature is a defining feature of the CSC model, whereby they undergo symmetric division to replenish the CSC pool and irreversible asymmetric division to generate daughter cells (non-CSCs) with low tumorigenic potential [36]. Tumorigenic cells can be distinguished from nontumorigenic cells based on marker expression by the CSCs [35]. Both proponents argue that the tumor microenvironment substantially influences the processes of carcinogenesis and tumor progression [31].

The plastic CSC theory, a third and evolving model, states that bidirectional conversions exist between non-CSCs and CSCs [36]. According to this model, the missing link between the two conventional models is that non-stem cells and non-CSCs can undergo a dedifferentiation process and reenter the stem cell/CSC state due to aberrant changes in gene expression [36]. Factors such as hepatocyte growth factor (HGF), CXC chemokine receptor-7 (CXCR7), and IL-6, which are derived from mesenchymal cells, contribute for the dedifferentiation [38]. Microenvironment specific for the individual tumor affects the plasticity-driven CSC niche, and an understanding of this phenomenon is critical for developing a more effective cancer treatment [39].

## 4. Implications of Intratumoral Heterogeneity for Cancer Treatment

Intratumoral heterogeneity arises due to complex genetic, epigenetic, and protein modifications resulting in phenotypic selection in response to environmental insult. This feature gives the tumor significant adaptability to thrive under unfamiliar conditions such as hypoxia or chemical weaponry [40]. Cell-to-cell variability, either genetic or not, can compromise responses to cancer therapies by increasing the repertoire of possible cellular responses [33]. One of the clinical implications of intratumoral heterogeneity is drug resistance and treatment failure [33]. Therefore, identification of intratumoral heterogeneity, which represents genetic characteristics of different cell subpopulations within the primary tumor, could provide important clinical implications to overcome this considerable challenge [41]. CSCs and interaction with their nurturing microenvironment (niche) were implicated as potential mechanisms underlying the intratumoral heterogeneity [42], and destroying these tumor microenvironments is recently considered to be one of the therapeutic targets against CSCs [43]. The hypothetical implication of this heterogeneity on therapeutic approach may include either concurrent combination or sequential treatments with multiple mutation-targeting agents [33]. Targeting these breast CSCs with therapeutics could be instrumental to achieve durable clinical responses. Therefore, expanded understanding of biology of breast CSCs and their key signaling pathways, molecular diagnosis of breast tumors, and identification of appropriate clinical trial endpoints are essential for the development of CSC targeting agents [44].

## 5. Biology and Biomarkers of Breast CSCs

Stem cells are undifferentiated cells defined by their properties of self-renewal and potency and are rare in nature [45]. Breast cancers contain CSCs, and these cells are thought to be involved in tumor initiation, progression, evolution, and metastasis [46]. CSCs were first identified in acute myeloid leukemia in 1994 and are defined by their unlimited self-renewal ability and their capacity to initiate and maintain malignancy [47, 48]. CSCs, typically constituting 1–5 % (could be as high as 11–35 % in breast cancer) of the tumor size, are tumorigenic multipotential cells with dysregulated self-renewal properties in which upon division, one daughter cell retains stemness and the other becomes committed to a lineage [49]. Characteristically, these cells are slow-dividing and have a lower ability to undergo apoptosis and a higher ability of DNA repair [50]. Breast CSCs are the best characterized subpopulations being the first CSCs prospectively demonstrated in human solid tumors [51]. Correlation between epithelial-to-mesenchymal transition (EMT) and CSCs was reported, and CSCs displaying mesenchymal characteristics are resistant to chemo- and radiotherapy and are considered responsible for recurrence of the disease after treatment [52, 53]. The EMT promotes cancer cell migration and invasion resulting in the reconstitution of metastatic colonies at distant sites [54].

Phenotypic markers including EPCAM/ESA^+^ (epithelial cell adhesion molecule/epithelial specific antigen), CD44^+^, CD24^−^, CD90, CD133, and *α*6-integrin [51, 55] and hedgehog-GLI and high aldehyde dehydrogenase (ALDH+) activity [56] best characterize breast CSCs. Cells rich with CD44^+^/CD24^−^/ALDH^+^ markers were reported to be more tumorigenic, metastatic, invasive, more migratory, and associated with poor clinical outcome and decreased patient survival [57, 58].

## 6. CSC Signaling Pathways as a Potential Target for Cancer Treatment

**Figure 1:**
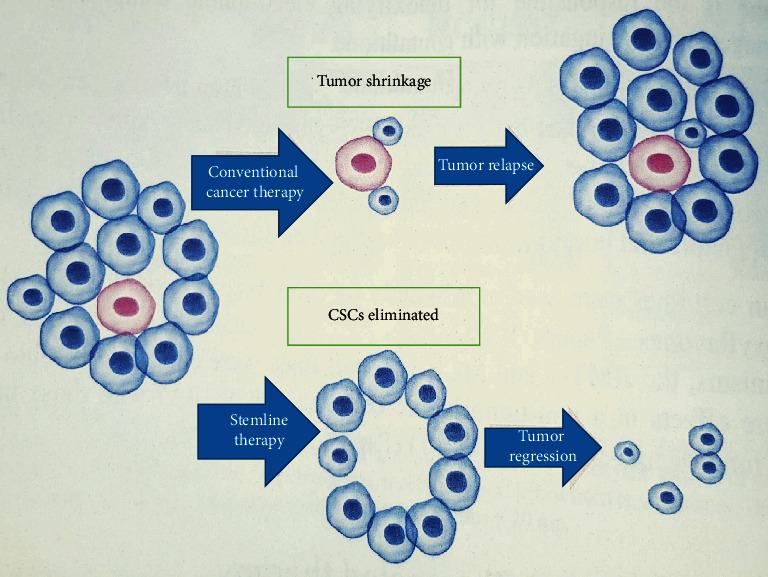
Graphic elucidation of the theory of CSC-based therapeutics. The CSCs (pink) are resistant to conventional therapeutics than their nontumorigenic counterparts (blue). The bulk tumor initially shrinks by the conventional therapeutics, though it repopulates by the virtue of treatment-resistant CSCs. If the CSCs are eradicated, on the other hand, the tumor regresses and eventually disappears.

Conventional therapies against cancer have multiple limitations that lead to treatment failure and cancer recurrence (Figure 1) [59]. Dysregulation of signal pathway network plays an important role in retaining the stemness of CSCs [60] and thus can possibly be eradicated by targeted therapeutics against those signaling pathways. The signaling pathways which are crucial for the biological functions of normal stem cells are abnormally activated or repressed in CSCs. Distinct and specific surface biomarker phenotypes and upregulated intracellular features can be used to distinguish CSCs from normal stem cells [60]. Seemingly, in addition, CSCs have their own specific enhanced signaling pathways [61]. They are also protected against xenobiotics by the high expression of ATP-binding cassette (ABC) transporter proteins, the characteristic feature that differentiates the CSCs from normal cells. Targeting these transporter proteins can be one of the key strategies to overcome resistance to chemotherapy [62, 63].

Breast CSCs exhibit high levels of Wnt, Notch, Hedgehog, JAK/STAT, PTEN, PI3K/Akt, NF-*κ*B, and ROS activity. These pathways play fundamental roles in maintaining self-renewal capacity of the CSCs [64]. Depending on these aberrant pathways, breast CSCs acquire their unique ability to initiate carcinoma and promote recurrence even after surgery [60]. Therefore, these pathways could be employed for the selective and targeted breast CSC therapeutics for better prognosis, patient care, and lasting cure.

### 6.1. Wnt/*β*-Catenin Signaling Pathway

Wints are secreted, cysteine-rich glycoproteins that act as short-range ligands. Its name is derived by joining the name of the Drosophila segment polarity gene “wingless” and the name of the vertebrate homolog, “int-1” [65]. Wnt/*β*-catenin pathway is initiated by evolutionarily conserved growth factors of Wnts encoded by 19 different Wnt genes and shares a high degree of sequence homology [66]. The canonical Wnt signaling cascade, also referred to as *β*-catenin-dependent Wnt pathway, is initiated by the binding of secreted Wnt proteins to a receptor complex consisting of a member of the Frizzled (FZD) family (seven-pass transmembrane receptor) [67]. Co-receptors like low-density lipoprotein receptor-related proteins 5 or 6 (LRP5/6) or tyrosine kinase-like orphan receptor 2 (ROR2) aid the binding of Wnt proteins to the receptor [68]. In the absence of Wnt signaling, the cytoplasmic *β*-catenin is maintained at a low level through ubiquitin-proteasome-mediated degradation and is regulated by a multiprotein destruction complex comprising Axin, adenomatous polyposis coli (APC), and glycogen synthase kinase-3*β* (GSK-3*β*) [69]. During the signal transduction, the adaptor protein Dishevelled (DVL) is phosphorylated and interacts with proteins in the complex and inhibits enzymatic activity of GSK3*β* within the destruction complex [69]. This action promotes unphosphorylated *β*-catenin accumulation and translocation into the nucleus, where it interacts with transcription factors to activate transcription of Wnt target genes, including cyclin D1 (CCND1), T-cell factor/lymphoid enhancer-binding factor (LEF1), and fibronectin (FN1) [70].

Abnormal activation of this pathway has been identified in breast cancer and is associated with the maintenance of CSC properties [71, 72], and this aberrant activation has not yet been clearly understood [69]. Mutations, epigenetic changes, or cells in the tumor microenvironment have been implicated, however [42]. Increase in cell motility due to its activation [73] and suppression of aggressive behavior by blocking the pathway were also noted [72]. Aberrant *β*-catenin expression was associated with poor clinical outcome in advanced breast cancer, especially basal and TNBC subtypes [74]. Hence, the inhibition of Wnt signaling pathway could be a potential therapeutic strategy to target CSCs and the high *β*-catenin expression can be used as a biomarker of advanced breast cancer.

### 6.2. Notch Signaling Pathway

Notch signaling, an evolutionarily conserved pathway, is composed of mammalian transmembrane receptors (notch 1–4) and their membrane-bound ligands (JAG1, JAG2, and *δ*-like ligand 1, 3, and 4 from Delta/Serrate/LAG-2 (DSL) families) [75]. Notch signaling requires a direct cell-cell contact allowing for short-range communication [76], and the pathway is, hence, ideally suited to control self-renewal and differentiation in stem cell microenvironment [77]. It is the pathway involved in the development of the breast and is frequently dysregulated in invasive breast cancer [78]. There is high expression of notch1 in basal-like 1 and mesenchymal-stem-like TNBCs, and positive correlation is established between high expression of notch1 and its ligand JAG1 and poor prognosis [79]. It is also responsible for the crucial steps of the EMT and angiogenesis [80]. EMT is mediated, in part, by SNAI1 (snail) and SNAI2 (slug) genes, two transcription repressors of E-cadherin that are known to be targets of the notch signaling pathway, and JAG1-induced notch activation increases EMT [81].

In a notch signaling, a ligand should carry out a receptor proteolysis in order to release an active notch fragment. The transduction of this pathway is activated by ligand binding, followed by a cleavage by metalloprotease and *γ*-secretase enzymes that produce the notch intracellular domain (NICD), a co-activator of transcription through inhibition of transcriptional repressors [82]. Upon translocation to the nucleus, NICD induces proliferation by directly activating myc, cyclin D1, and CDK5 [83]. In breast CSCs, the relationships between notch deregulated signaling and the carcinogenesis process reinforced by notch crosstalk with many oncogenic signaling pathways suggest that notch signaling may be a critical drug target for breast cancer [84].

### 6.3. Hedgehog Signaling Pathway

Hedgehog signaling involves a complex network of molecules. Its transduction is effected by canonical or noncanonical mechanisms. Canonically, it is initiated by three glycoprotein ligands: Desert hedgehog (DHH), Indian hedgehog (IHH), or Sonic hedgehog (SHH) which bind to the 12-pass transmembrane protein receptors Patched (PTCH), located on the plasma membrane [85]. The aberrant activation of the pathway is effected through the overexpression of hedgehog ligands, loss of function of the receptor, or dysregulation of the transcription factors [85, 86]. All these aberrations have been implicated in initiation and progression of breast cancer [87].

The PTCH receptor sequesters a protein smoothened (SMO), a 7-pass transmembrane G-protein coupled signal transduction molecule, in a cytoplasm in the absence of hedgehog ligands. Glioma associated oncogene homolog proteins (GLI) are also sequestered in the cytoplasm by forming a large complex with SUFU (suppressor of fused) and KIF7 (kinesin family member 7). These inhibitors block PKA (protein kinase A), CK1 (casein kinase 1), and GSK-3*β* (glycogen synthase kinase-3*β*) from phosphorylating the GLI proteins, maintaining its repressive form. When the ligand binds to PTCH, PTCH-mediated inhibition of the SMO complex is relieved and signal transduction is initiated in the receiving cell resulting in the translocation of the GLI transcription factors to the nucleus. Its translocation results in modulation of the transcription of genes such as Fox, Myc, and cyclin D among others, and the downstream effects include proliferation, differentiation, epithelial-to-mesenchymal transition, metastasis, and maintaining the stemness [85, 87–89].

### 6.4. NF-*κ*B Signaling Pathway

NF-*κ*B (nuclear factor-kappa B) refers to a family of transcription factors that control multiple cellular processes in cancer including inflammation, transformation, proliferation, survival, angiogenesis, invasion, metastasis, chemoresistance, and radioresistance [90]. It is also implicated to regulate the transcription of EMT transcription factor genes in breast cancer [91]. The NF-*κ*B family consists of five transcription factors: RelA (p65), RelB, c-Rel, p100/p52 (NF-*κ*B2) (p100 is a larger precursor from which p52 is derived), and p105/p50 (NF-*κ*B1) (p105 is a larger precursor from which p50 is derived); these factors can homo- or heterodimerize to form active transcription factors [92]. The p65 (RelA), RelB, and c-Rel proteins harbor a C-terminal transactivation domain (TAD) that interacts with the transcription machinery that promotes gene transcription, whereas the homodimers of p50 or p52 lack a TAD and serve as transcription repressors that provide a threshold for NF-*κ*B activation [93].

The principle inactive form of the NF-kB complex is found primarily located in the cytoplasm associated with I*κ*B proteins, inhibitor of NF-*κ*B, as a p50–p65–IkB*α* trimer [90]. For the NF-*κ*B complex to be released from its inhibitor, the I*κ*B protein must be phosphorylated by the I*κ*B kinase (IKK) complex, which leads to I*κ*B ubiquitination (addition of ubiquitin—a signal for degradation) and subsequent degradation by the proteasome. NF-*κ*B is then translocated into the nucleus and activates gene transcription by binding to sequence-specific target DNA [94].

For activation of NF-kB by canonical and noncanonical pathways, the regulatory kinase is IKK, which is a complex of three proteins, two catalytic (IKK*α* and IKK*β*) and one regulatory (IKK*γ*, also known as the NF-kB essential modulator (NEMO)) [90]. Induction of phosphorylation by signals and subsequent degradation of NF-*κ*B inhibitors (I*κ*B proteins) is basis for the activation, both canonically and noncanonically. After degradation of NF-*κ*B inhibitors, the NF-*κ*B pathway is canonically activated by translocation of NF-*κ*B dimmers (p50:p65 dimer) [92]. The canonical pathway is the major pathway in most cell types and it involves p65, c-Rel, and p50 [95]. This pathway consists of IKK (an I*κ*B kinase heterodimer), I*κ*B, and NF-*κ*B (typically a p65/p50 heterodimer) [96]. Proinflammatory cytokines, IL-1*β* and TNF-*α*, and variety of cellular stresses were found to activate the pathway [93].

In breast cancer, activation of NF-*κ*B is one of the frequent features of chronic inflammation [97]. In addition, the constitutive activation of NF-*κ*B in almost all breast cancer forms shows its oncogenic feature and reveals its association with initiation and progression of breast cancer [98]. Aberrant NF-*κ*B activation has been shown to be involved in breast CSC phenotypic features by cross-talking to several other signaling pathways [90]. The transcriptional activation of genes associated with cell proliferation, angiogenesis, metastasis, and suppression of apoptosis appears to lie at the heart of the ability of NF-*κ*B to promote oncogenesis and cancer therapy resistance; targeting this pathway is, therefore, of great therapeutic importance against breast CSCs [99].

### 6.5. JAK/STAT Signaling Pathway

The Janus kinase (JAK)/signal transducer and activator of transcription (STAT) pathway was originally identified as an intracellular signaling pathway mediating cytokine signals [100], and there are seven STAT proteins (STAT1-4, 5A, 5B and 6) and four JAK kinases (JAK1–3 and tyrosine kinase 2 (TYK2)) in mammals [101]. Canonical signaling is based on STAT tyrosine phosphorylation by activated JAKs. Downstream of interferon (IFN) receptors, activated JAKs cause the formation of the transcription factors IFN-stimulated gene factor 3 (ISGF3), a heterotrimer of STAT1, STAT2, and interferon regulatory factor 9 (IRF9) subunits, and gamma interferon-activated factor (GAF), a STAT1 homodimer [102]. Precisely, binding of the protein to the receptor induces dimerization, which activates the associated JAKs [79]. The JAKs also phosphorylate STATs, which lead to their dimerization, nuclear translocation, and transcriptional regulation of genes [103]. It has widely been assumed that the effects of STAT activation are mediated by direct transcriptional induction of STAT target genes, providing a mechanism for transcriptional regulation without second messengers [100, 104]. Aberrant JAK/STAT signaling pathway has been identified to contribute to cancer progression and metastatic development [104]. There is emerging preclinical evidence that disruption of the JAK2/STAT3 signaling could be an effective clinical strategy to treat breast cancer, especially TNBC [103].

### 6.6. PI3K/Akt Signaling Pathway

The phosphatidylinositol 3-kinase (PI3Ks) pathway comprises a family of intracellular signal transducer enzymes—PI3K, Akt (serine/threonine kinase), and mammalian (or mechanistic) target of rapamycin (mTOR) [105]. It is activated by the binding of a growth factor or ligand to its cognate growth factor receptor tyrosine kinases (RTKs) (e.g., HER, insulin, and insulin-like growth factor 1 (IGF-1) receptor) [106].The pathway mediates complicated multiple cellular processes including cell survival, metabolism, proliferation, motility, migration, invasion, and angiogenesis [79]. It also confers resistance to conventional therapies and results in poor breast cancer prognosis [107]. PI3K/Akt pathway is deregulated by gain- or loss-of-functional mutations in the tumor development of various cancer types, and it activates a number of oncogenic pathways in breast cancer and is implicated in cellular transformation and tumorigenesis [108]. Akt pathway is one of the promising targets of therapy in breast cancer [105], and several inhibitors including PI3K inhibitors, Akt inhibitors, mTOR catalytic site inhibitors, and dual PI3K-mTOR inhibitors have already been designed and few are under clinical trials [109].

### 6.7. PTEN Signaling Pathway

PTEN (phosphatase and tensin homolog) plays an important role in tumor suppression by negatively regulating the oncogenic phosphatidylinositol 3-kinase (PI3K) pathway [110]. It plays an important role in the regulation of cell growth and apoptosis, and it is dysregulated in breast cancer, often [111]. Though the mechanism by which PTEN is downregulated is poorly understood, mutations, copy number loss, rearrangements, epigenetic silencing, and posttranslational regulation may contribute [112]. Somatic loss-of-function mutations of PTEN in sporadic breast carcinomas are estimated to be about 30–40 % and are found across the entire spectrum of tumor types [113]. It is also implicated in breast cancer progression and resistance to targeted therapies and is thought to promote tumorigenesis by activating PI3K signaling [114]. Of note, permanent PI3K/Akt pathway activation was associated with the loss of PTEN activity [115]. PTEN has been reported to be targeted by many miRNAs, and MiRNA-21, for instance, induces EMT via the PTEN/Akt pathway in breast cancer [111]. Since PTEN is dedicated to inhibit the PI3K-Akt pathway, targeting PTEN aims to control an aberrant activation of oncogenic PI3K-Akt pathway, a major survival pathway activated in breast cancer [116].

### 6.8. Intracellular ROS Signaling

Reactive oxygen species (ROS) are crucial role players in various biological functions including tissue homeostasis, differentiation, cellular signaling, and survival. In cancer, its location, local concentration, and type of the ROS generated are important determinants for its cellular functions. Due to high metabolic rate, gene mutation, and relative hypoxia, the production of ROS is enhanced in CSCs and this causes this subpopulation to differentiate, become senescent, and commit an apoptosis [117, 118]. However, CSCs are known to contain lower levels of ROS attributable to the increased production of free radical scavengers, when compared to the nontumorigenic breast cancer mass. The lower ROS level is associated with maintaining the stemness of the CSCs and is accounted for resistance to radiotherapy [118–120]. For novel breast cancer therapeutic strategies, this feature poses unprecedented challenge. The ROS are required to early events in tumor development. On the other hand, lower levels of ROS are required to CSCs maintenance. Therefore, identification of cancer cell-specific ROS-sensing signaling pathways mediating the diverse stress-regulated cellular functions is indispensable to curb this challenge [120].

## 7. Emerging Therapeutic Frontiers

### 7.1. Nanomedicine

Nanomedicine has revolutionized drug delivery, allowing the therapeutic agents to selectively targeting CSCs and minimizing toxicity to normal cells [121]. Nanocarriers (e.g., polymeric and metal nanoparticles, polymeric micelles, liposomes, and carbon nanotubes) possess the properties of high drug loading capacity, solubility enhancement effects, site-specific delivery mechanism, negligible release of drug prematurely, and controlled release mechanism that provides effective drug doses to the target site [43, 122]. During conventional breast cancer drug delivery, major limitations include offsite effects, instability, poor solubility, short circulation half-life, undesirable bioavailability, and poor cellular uptake among others. Though further efforts are needed, advancements in nanomedicine will help curb these hurdles [123].

### 7.2. Role of miRNAs in Diagnostics and Therapeutics

MiRNAs are endogenous, small noncoding RNA molecules that are approximately 20–22 nucleotides long. They exert their gene-regulatory function on the posttranscriptional level via inhibition of mRNA translation or degradation, depending on the complementarity to their mRNA target [104]. Depending on the target gene regulated, miRNAs can either serve as tumor suppressor miRNAs by repressing oncogenes or “OncomiRNAs” by targeting tumor suppressor genes [124]. In breast cancer, a number of miRNAs have been identified as tumor suppressors or oncogenes and have been characterized as critical regulators of tumor initiation, metastasis, and chemoresistance [125]. Dysregulation of oncogenic miRNA-21 and miRNA-125b, for instance, was found to promote tumor growth by inhibiting apoptosis pathways, allowing cell cycle progression, stimulating tumor proliferation, and promoting invasion and metastasis [126]. MiRNA-155 was overexpressed in breast cancer and downregulated the repressor SOCS1 (suppressor of cytokine signaling 1), resulting in an aberrant activation of STAT3 signaling pathway [127]. MiRNAs have cancer-specific expression profiles and can be used in tracing cancer origin of metastasis. In addition to their significance as therapeutic targets, they are important diagnostic and therapeutic response biomarkers [124].

## 8. Prospective Remarks

As new technologies allow for easier descriptions of genetic, epigenetic, and environmental chemical exposure profiles of individual tumors, the number and specificity of tumor classifications will inevitably increase substantially. Single cell sequencing and analysis of cell-free DNA might broaden the means to understand intratumoral heterogeneity with greater precision and would further help to overcome the diagnostic and therapeutic challenges [44]. Differences in gene expression among patients with the same subtype of breast cancer are correlated with the response to treatment. This strongly suggests a personalized approach to treat breast cancer [128]. This complicated therapeutic approach requirement would further necessitate targeting CSCs as a root cause of the problem. Though not effective to date, attempts to reactivate tumor cell death pathways to induce cancer regression are also among the current anticancer strategies, and it could augment CSC-based strategies for a lasting breast cancer cure [129].

## Abbreviations

AKT: Serine/threonine kinase (also called protein kinase B)
ALDH: Aldehyde dehydrogenase
CK1: Casein kinase 1
CSCs: Cancer stem cells
DSL: Delta/Serrate/LAG-2
EMT: Epithelial-to-mesenchymal transition
ER: Estrogen receptor
ERBB2: Erb-b2 receptor tyrosine kinase 2
GLI: Glioma associated oncogene homolog
GSK-3*β*: Glycogen synthase kinase-3*β*
IDC-NST: Invasive ductal carcinomas of no special type
IKK: Inhibitor of kappa light polypeptide gene enhancer in B-cells, kinase
I*κ*B*α*: NF-*κ*B inhibitor, *α*
JAK: Janus kinase
KIF7: Kinesin family member 7
mTOR: Mammalian (or mechanistic) target of rapamycin
NEMO: NF-*κ*B essential modulator
NF-*κ*B: Nuclear factor kappa-light-chain-enhancer of activated B-cells
NICD: Notch intracellular domain
P53/TP53: Tumor protein 53
PI3Ks: Phosphatidylinositol 3-kinase
PKA: Protein kinase A
PR: Progesterone receptor
PTCH: 12-pass transmembrane protein receptors Patched
PTEN: Phosphatase and tensin homolog
ROS: Reactive oxygen species
RTK: Receptor tyrosine kinase
SMO: Smoothened protein encoded by SMO gene
STAT: Signal transducer and activator of transcription
SUFU: Suppressor of fused
TAD: C-terminal transactivation domain
TCGA: Cancer Genome Atlas
TNBC: Triple negative breast cancers
TNF: Tumor necrosis factor
WHO: World Health Organization.

## References

[1] Long J. S., Ryan K. M. (2012). New frontiers in promoting tumour cell death: targeting apoptosis, necroptosis and autophagy. *Oncogene*.

[2] Font-Burgada J., Sun B., Karin M. (2016). Obesity and cancer: the oil that feeds the flame. *Cell Metabolism*.

[3] Bray F., Ferlay J., Soerjomataram I., Siegel R. L., Torre L. A., Jemal A. (2018). Global cancer statistics 2018: GLOBOCAN estimates of incidence and mortality worldwide for 36 cancers in 185 countries. *CA: A Cancer Journal for Clinicians*.

[4] Torre L. A., Bray F., Siegel R. L. (2015). Global cancer statistics, 2012. *CA: A Cancer Journal for Clinicians*.

[5] WHO (Febuary 2019). Fact sheet no. 297 cancer. http://www.who.int/mediacentre/factsheets/fs297/en.

[6] World Health Organization (Febuary 2019). http://www.who.int/news-room/fact-sheets/detail/cancer.

[7] Torre L. A., Siegel R. L., Ward E. M., Jemal A. (2016). Global cancer incidence and mortality rates and trends-an update. *Cancer Epidemiology Biomarkers & Prevention*.

[8] Levitsky D. O., Dembitsky V. M. (2015). Anti-breast cancer agents derived from plants. *Natural Products and Bioprospecting*.

[9] Burney I. A., Furrukh M., Al-Moundhri M. S. (2014). What are our options in the fight against breast cancer?. *Sultan Qaboos University Medical Journal*.

[10] WHO (2019). *Breast Cancer: Prevention and Control*.

[11] Tfayli A., Temraz S., Abou Mrad R., Shamseddine A. (2010). Breast cancer in low- and middle-income countries: an emerging and challenging epidemic. *Journal of Oncology*.

[12] Minisini A. M., Moroso S., Gerratana L. (2013). Risk factors and survival outcomes in patients with brain metastases from breast cancer. *Clinical & Experimental Metastasis*.

[13] Tao Z., Shi A., Lu C., Song T., Zhang Z., Zhao J. (2015). Breast cancer: epidemiology and etiology. *Cell Biochemistry and Biophysics*.

[14] May F. (2014). Novel drugs that target the estrogen-related receptor alpha: their therapeutic potential in breast cancer. *Cancer Management and Research*.

[15] Facts for life: What is breast cancer? January 2017, http://www.komen.org

[16] Chlebowski R. T., Manson J. E., Anderson G. L. (2013). Estrogen plus progestin and breast cancer incidence and mortality in the women’s health initiative observational study. *JNCI: Journal of the National Cancer Institute*.

[17] Jacobs E. T., Kohler L. N., Kunihiro A. G., Jurutka P. W. (2016). Vitamin D and colorectal, breast, and prostate cancers: a review of the epidemiological evidence. *Journal of Cancer*.

[18] Spivey A. (2010). Light pollution: light at night and breast cancer risk worldwide. *Environmental Health Perspectives*.

[19] Clagett O., Plimpton N., Root G. (2016). Lesions of the breast: the relationship of benign lesions to carcinoma. *Surgery*.

[20] White A. J., Bradshaw P. T., Herring A. H. (2016). Exposure to multiple sources of polycyclic aromatic hydrocarbons and breast cancer incidence. *Environment International*.

[21] Engmann N. J., Golmakani M. K., Miglioretti D. L., Sprague B. L., Kerlikowske K. (2017). Population-attributable risk proportion of clinical risk factors for breast cancer. *JAMA Oncology*.

[22] Warner E. T., Hu R., Collins L. C. (2016). Height and body size in childhood, adolescence, and young adulthood and breast cancer risk according to molecular subtype in the nurses’ health studies. *Cancer Prevention Research*.

[23] Parada H., Steck S. E., Bradshaw P. T. (2017). Grilled, barbecued, and smoked meat intake and survival following breast cancer. *Journal of the National Cancer Institute*.

[24] Belpomme D., Irigaray P., Sasco A. (2007). The growing incidence of cancer: role of lifestyle and screening detection (review). *International Journal of Oncology*.

[25] Jemal A., Bray F., Center M. M., Ferlay J., Ward E., Forman D. (2011). Global cancer statistics. *CA: A Cancer Journal for Clinicians*.

[26] Ginsburg O. M. (2013). Breast and cervical cancer control in low and middle-income countries: human rights meet sound health policy. *Journal of Cancer Policy*.

[27] Baburin A., Aareleid T., Rahu M., Reedik L., Innos K. (2016). Recent changes in breast cancer incidence and mortality in Estonia: transition to the west. *Acta Oncologica*.

[28] Giordano S. H. (2005). A review of the diagnosis and management of male breast cancer. *The Oncologist*.

[29] Fentiman I., Fourquet A., Hortobagyi G. (2009). Male breast cancer: a review. *Ecancermedicalscience*.

[30] Weiss J. R., Moysich K. B., Swede H. (2005). Epidemiology of male breast cancer. *Cancer Epidemiology, Biomarkers & Prevention*.

[31] Hsiao Y.-H., Chou M.-C., Fowler C., Mason J. T., Man Y.-G. (2010). Breast cancer heterogeneity: mechanisms, proofs, and implications. *Journal of Cancer*.

[32] Verma R., Bowen R., Slater S. (2012). Pathological and epidemiological factors associated with advanced stage at diagnosis of breast cancer. *British Medical Bulletin*.

[33] Sun X.-x., Yu Q. (2015). Intra-tumor heterogeneity of cancer cells and its implications for cancer treatment. *Acta Pharmacologica Sinica*.

[34] Cho N. (2016). Molecular subtypes and imaging phenotypes of breast cancer. *Ultrasonography*.

[35] Shackleton M., Quintana E., Fearon E. R., Morrison S. J. (2009). Heterogeneity in cancer: cancer stem cells versus clonal evolution. *Cell*.

[36] Marjanovic N. D., Weinberg R. A., Chaffer C. L. (2013). Cell plasticity and heterogeneity in cancer. *Clinical Chemistry*.

[37] Yang T., Rycaj K., Liu Z.-M., Tang D. G. (2014). Cancer stem cells: constantly evolving and functionally heterogeneous therapeutic targets. *Cancer Research*.

[38] Medema J. P. (2013). Cancer stem cells: the challenges ahead. *Nature Cell Biology*.

[39] Lee G., Hall R. R., Ahmed A. U. (2016). Cancer stem cells: cellular plasticity, niche, and its clinical relevance. *Journal of Stem Cell Research & Therapy*.

[40] Y-Cajal S. R., Sesé M., Capdevila C. (2020). Clinical implications of intratumor heterogeneity: challenges and opportunities. *Journal of Molecular Medicine*.

[41] Ziogas D. E., Spiliotis J., Lykoudis E. G., Zografos G. C., Roukos D. H. (2017). Intratumor and circulating clonal heterogeneity shape the basis of precision breast cancer therapy. *Future Oncology*.

[42] Korkaya H., Liu S., Wicha M. S. (2011). Breast cancer stem cells, cytokine networks, and the tumor microenvironment. *Journal of Clinical Investigation*.

[43] Shen S., Xia J.-X., Wang J. (2016). Nanomedicine-mediated cancer stem cell therapy. *Biomaterials*.

[44] Pareja F., Marchiò C., Geyer F. C., Weigelt B., Reis-Filho J. S. (2017). Breast cancer heterogeneity: roles in tumorigenesis and therapeutic implications. *Current Breast Cancer Reports*.

[45] Li M., Cascino P., Ummarino S., Di Ruscio A. (2017). Application of induced pluripotent stem cell technology to the study of hematological diseases. *Cells*.

[46] Ahmed M., Chaudhari K., Babaei-Jadidi R., Dekker L. V., Shams Nateri A. (2017). Concise review: emerging drugs targeting epithelial cancer stem-like cells. *Stem Cells*.

[47] Lapidot T., Sirard C., Vormoor J. (1994). A cell initiating human acute myeloid leukaemia after transplantation into SCID mice. *Nature*.

[48] Relation T., Dominici M., Horwitz E. M. (2017). Concise review: an (Im) penetrable shield: how the tumor microenvironment protects cancer stem cells. *Stem Cells*.

[49] Nigam A. (2013). Breast cancer stem cells, pathways and therapeutic perspectives 2011. *Indian Journal of Surgery*.

[50] Morrison B. J., Schmidt C. W., Lakhani S. R. (2008). Breast cancer stem cells: implications for therapy of breast cancer. *Breast Cancer Research*.

[51] Al-Hajj M., Wicha M. S., Benito-Hernandez A., Morrison S. J., Clarke M. F. (2003). Prospective identification of tumorigenic breast cancer cells. *Proceedings of the National Academy of Sciences*.

[52] Wu Y., Sarkissyan M., Vadgama J. V. (2016). Epithelial-mesenchymal transition and breast cancer. *Journal of Clinical Medicine*.

[53] Shah M., Allegrucci C. (2012). Keeping an open mind: highlights and controversies of the breast cancer stem cell theory. *Breast Cancer: Targets and Therapy*.

[54] Bill R., Christofori G. (2015). The relevance of EMT in breast cancer metastasis: correlation or causality?. *FEBS Letters*.

[55] Horimoto Y., Arakawa A., Sasahara N. (2016). Combination of cancer stem cell markers CD44 and CD24 is superior to ALDH1 as a prognostic indicator in breast cancer patients with distant metastases. *PloS One*.

[56] Ginestier C., Hur M. H., Charafe-Jauffret E. (2007). ALDH1 is a marker of normal and malignant human mammary stem cells and a predictor of poor clinical outcome. *Cell Stem Cell*.

[57] Velasco-Velázquez M. A., Popov V. M., Lisanti M. P., Pestell R. G. (2011). The role of breast cancer stem cells in metastasis and therapeutic implications. *The American Journal of Pathology*.

[58] Marcato P., Dean C. A., Pan D. (2011). Aldehyde dehydrogenase activity of breast cancer stem cells is primarily due to isoform ALDH1A3 and its expression is predictive of metastasis. *Stem Cells*.

[59] Li X., Lewis M. T., Huang J. (2008). Intrinsic resistance of tumorigenic breast cancer cells to chemotherapy. *JNCI Journal of the National Cancer Institute*.

[60] Chen K., Huang Y.-H., Chen J.-L. (2013). Understanding and targeting cancer stem cells: therapeutic implications and challenges. *Acta Pharmacologica Sinica*.

[61] Pardal R., Clarke M. F., Morrison S. J. (2003). Applying the principles of stem-cell biology to cancer. *Nature Reviews Cancer*.

[62] Fletcher J. I., Haber M., Henderson M. J., Norris M. D. (2010). ABC transporters in cancer: more than just drug efflux pumps. *Nature Reviews Cancer*.

[63] Sridharan S., Howard C. M., Tilley A. M. (2019). Novel and alternative targets against breast cancer stemness to combat chemoresistance. *Frontiers in Oncology*.

[64] Hinohara K., Gotoh N. (2010). Inflammatory signaling pathways in self-renewing breast cancer stem cells. *Current Opinion in Pharmacology*.

[65] Niehrs C. (2012). The complex world of WNT receptor signalling. *Nature Reviews Molecular Cell Biology*.

[66] Clevers H., Nusse R. (2012). Wnt/*β*-Catenin signaling and disease. *Cell*.

[67] Dandawate P. R., Subramaniam D., Jensen R. A. Targeting cancer stem cells and signaling pathways by phytochemicals: novel approach for breast cancer therapy.

[68] Rosso S. B., Inestrosa N. C. (2013). WNT signaling in neuronal maturation and synaptogenesis. *Frontiers in Cellular Neuroscience*.

[69] Kazi M. M., Trivedi T. I., Kobawala T. P. (2016). The potential of Wnt signaling pathway in cancer: a focus on breast cancer. *Cancer Translational Medicine*.

[70] MacDonald B. T., Tamai K., He X. (2009). Wnt/*β*-Catenin signaling: components, mechanisms, and diseases. *Developmental Cell*.

[71] Zeng Y. A., Nusse R. (2010). Wnt proteins are self-renewal factors for mammary stem cells and promote their long-term expansion in culture. *Cell Stem Cell*.

[72] Jang G.-B., Kim J.-Y., Cho S.-D. (2015). Blockade of Wnt/*β*-catenin signaling suppresses breast cancer metastasis by inhibiting CSC-like phenotype. *Scientific Reports*.

[73] Schlange T., Matsuda Y., Lienhard S., Huber A., Hynes N. E. (2007). Autocrine WNT signaling contributes to breast cancer cell proliferation via the canonical WNT pathway and EGFR transactivation. *Breast Cancer Research*.

[74] Khramtsov A. I., Khramtsova G. F., Tretiakova M., Huo D., Olopade O. I., Goss K. H. (2010). Wnt/*β*-Catenin pathway activation is enriched in basal-like breast cancers and predicts poor outcome. *The American Journal of Pathology*.

[75] Nowell C. S., Radtke F. (2017). Notch as a tumour suppressor. *Nature Reviews Cancer*.

[76] Kopan R., Ilagan M. X. G. (2009). The canonical notch signaling pathway: unfolding the activation mechanism. *Cell*.

[77] Perdigoto C. N., Bardin A. J. (2013). Sending the right signal: notch and stem cells. *Biochimica et Biophysica Acta (BBA) - General Subjects*.

[78] Farnie G., Clarke R. B. (2007). Mammary stem cells and breast cancer-role of notch signalling. *Stem Cell Reviews*.

[79] Lehmann B. D., Pietenpol J. A., Tan A. R. (2015). *Triple-negative Breast Cancer: Molecular Subtypes and New Targets for Therapy: American Society of Clinical Oncology Educational Book*.

[80] Garcia-Heredia J. M., Lucena-Cacace A., Verdugo-Sivianes E. M. (2017). The cargo protein MAP17 (PDZK1IP1) regulates the cancer stem cell pool activating the notch pathway by abducting NUMB. *Clinical Cancer Research*.

[81] Chen J., Imanaka N., Chen J., Griffin J. D. (2010). Hypoxia potentiates notch signaling in breast cancer leading to decreased E-cadherin expression and increased cell migration and invasion. *British Journal of Cancer*.

[82] Guo S., Liu M., Gonzalez-Perez R. R. (2011). Role of notch and its oncogenic signaling crosstalk in breast cancer. *Biochimica et Biophysica Acta (BBA) - Reviews on Cancer*.

[83] Haider S., Pollheimer J., Knöfler M. (2017). Notch signalling in placental development and gestational diseases. *Placenta*.

[84] Astudillo L., Capobianco A. J. A small molecule inhibitor of the notch transcriptional activation complex.

[85] Hanna A., Shevde L. A. (2016). Hedgehog signaling: modulation of cancer properies and tumor mircroenvironment. *Molecular Cancer*.

[86] Sachin G., Naoko T., Patricia L. (2010). Targeting the hedgehog pathway in cancer. *Therapeutic Advances in Medical Oncology*.

[87] Gupta S., Takebe N., LoRusso P. (2010). Review: targeting the hedgehog pathway in cancer. *Therapeutic Advances in Medical Oncology*.

[88] Rimkus T., Carpenter R., Qasem S., Chan M., Lo H.-W. (2016). Targeting the sonic hedgehog signaling pathway: review of smoothened and GLI inhibitors. *Cancers*.

[89] Ryu J. S., Raucher D. Inhibition of breast cancer stem cells by Hedgehog-inhibitory peptide conjugated with Elastin-like biopolymers.

[90] Chaturvedi M. M., Sung B., Yadav V. R., Kannappan R., Aggarwal B. B. (2011). NF-*κ*B addiction and its role in cancer: ‘one size does not fit all’. *Oncogene*.

[91] Pires B. R., Mencalha A. L., Ferreira G. M. (2017). NF-kappaB is involved in the regulation of EMT genes in breast cancer cells. *PloS One*.

[92] Vazquez-Santillan K., Melendez-Zajgla J., Jimenez-Hernandez L. E. (2016). NF-kappaΒ-inducing kinase regulates stem cell phenotype in breast cancer. *Scientific Reports*.

[93] Hayden M. S., Ghosh S. (2004). Signaling to NF- B. *Genes & Development*.

[94] Gilmore T. D., Garbati M. R. (2011). Inhibition of NF-*κ*B signaling as a strategy in disease therapy. *Current Topics in Microbiology and Immunology*.

[95] Brock T. G. (Febuary 2017). NF-*κ*B. https://www.caymanchem.com/Article/2174.

[96] Lin Y., Bai L., Chen W., Xu S. (2010). The NF-*κ*B activation pathways, emerging molecular targets for cancer prevention and therapy. *Expert Opinion on Therapeutic Targets*.

[97] Yamaguchi N., Ito T., Azuma S. (2009). Constitutive activation of nuclear factor-*κ*B is preferentially involved in the proliferation of basal-like subtype breast cancer cell lines. *Cancer Science*.

[98] Neil J. R., Schiemann W. P. (2008). Altered TAB1:I B kinase interaction promotes transforming growth factor -mediated nuclear factor- B activation during breast cancer progression. *Cancer Research*.

[99] Li F., Sethi G. (2010). Targeting transcription factor NF-*κ*B to overcome chemoresistance and radioresistance in cancer therapy. *Biochimica et Biophysica Acta*.

[100] Aaronson D. S., Horvath C. M. (2002). A road map for those who don’t know JAK-STAT. *Science*.

[101] Li W. X. (2008). Canonical and non-canonical JAK-STAT signaling. *Trends in Cell Biology*.

[102] Majoros A., Platanitis E., Kernbauer-Hölzl E., Rosebrock F., Müller M., Decker T. (2017). Canonical and non-canonical aspects of JAK-STAT signaling: lessons from interferons for cytokine responses. *Frontiers in Immunology*.

[103] Aittomäki S., Pesu M. (2014). Therapeutic targeting of the Jak/STAT pathway. *Basic & Clinical Pharmacology & Toxicology*.

[104] Pencik J., Pham H. T. T., Schmoellerl J. (2016). JAK-STAT signaling in cancer: from cytokines to non-coding genome. *Cytokine*.

[105] Mayer I. A., Arteaga C. L. (2016). The PI3K/AKT pathway as a target for cancer treatment. *Annual Review of Medicine*.

[106] Porta C., Paglino C., Mosca A. (2014). Targeting PI3K/Akt/mTOR signaling in cancer. *Frontiers in Oncology*.

[107] Saini K. S., Loi S., de Azambuja E. (2013). Targeting the PI3K/AKT/mTOR and Raf/MEK/ERK pathways in the treatment of breast cancer. *Cancer Treatment Reviews*.

[108] Carmona F. J., Montemurro F., Kannan S. (2016). AKT signaling in ERBB2-amplified breast cancer. *Pharmacology & Therapeutics*.

[109] Yang S. X., Polley E., Lipkowitz S. (2016). New insights on PI3K/AKT pathway alterations and clinical outcomes in breast cancer. *Cancer Treatment Reviews*.

[110] Chen Y., van de Vijver M. J., Hibshoosh H., Parsons R., Saal L. H. (2016). PTEN and NEDD4 in human breast carcinoma. *Pathology & Oncology Research*.

[111] Wu Z.-H., Tao Z.-H., Zhang J. (2016). MiRNA-21 induces epithelial to mesenchymal transition and gemcitabine resistance via the PTEN/AKT pathway in breast cancer. *Tumor Biology*.

[112] Cancer Genome Atlas Network (2012). Comprehensive molecular portraits of human breast tumors. *Nature*.

[113] Zhang H.-Y., Liang F., Jia Z.-L., Song S.-T., Jiang Z.-F. (2013). PTEN mutation, methylation and expression in breast cancer patients. *Oncology Letters*.

[114] Ebbesen S. H., Scaltriti M., Bialucha C. U. (2016). PTEN loss promotes MAPK pathway dependency in HER2/neu breast carcinomas. *Proceedings of the National Academy of Sciences*.

[115] Cheung L. W. T., Hennessy B. T., Li J. (2011). High frequency of PIK3R1 and PIK3R2 mutations in endometrial cancer elucidates a novel mechanism for regulation of PTEN protein stability. *Cancer Discovery*.

[116] Georgescu M.-M. (2010). PTEN tumor suppressor network in PI3K-Akt pathway control. *Genes & Cancer*.

[117] Zhou D., Shao L., Spitz D. R. (2014). Reactive oxygen species in normal and tumor stem cells. *Advances in Cancer Research*.

[118] Perillo B., Di Donato M., Pezone A. (2020). ROS in cancer therapy: the bright side of the moon. *Experimental & Molecular Medicine*.

[119] Diehn M., Cho R. W., Lobo N. A. (2009). Association of reactive oxygen species levels and radioresistance in cancer stem cells. *Nature*.

[120] Liou G.-Y., Storz P. (2010). Reactive oxygen species in cancer. *Free Radical Research*.

[121] He L., Gu J., Lim L. Y. (2016). Nanomedicine-mediated therapies to target breast cancer stem cells. *Frontiers in Pharmacology*.

[122] Estanqueiro M., Amaral M. H., Conceição J., Sousa Lobo J. M. (2015). Nanotechnological carriers for cancer chemotherapy: the state of the art. *Colloids and Surfaces B: Biointerfaces*.

[123] He L., Gu J., Lim L. Y., Yuan Z.-X., Mo J. (2016). Nanomedicine-mediated therapies to target breast cancer stem cells. *Frontiers in Pharmacology*.

[124] Muluhngwi P., Klinge C. M. (2017). Identification of miRNAs as biomarkers for acquired endocrine resistance in breast cancer. *Molecular and Cellular Endocrinology*.

[125] Wu Q., Wang C., Lu Z., Guo L., Ge Q. (2012). Analysis of serum genome-wide microRNAs for breast cancer detection. *Clinica Chimica Acta*.

[126] Baranwal S., Alahari S. K. (2010). miRNA control of tumor cell invasion and metastasis. *International Journal of Cancer*.

[127] Jiang S., Zhang H. W., Lu M. H. (2010). MicroRNA-155 functions as an OncomiR in breast cancer by targeting the suppressor of cytokine signaling 1 gene. *Cancer Research*.

[128] Jhan J., Andrechek E. (2017). Effective personalized therapy for breast cancer based on predictions of cell signaling pathway activation from gene expression analysis. *Oncogene*.

[129] Manjari D., Dimri G. P., Coleman W. B., Tsongalis G. J. (2017). Senescence, apoptosis, and cancer. *The Molecular Basis of Human Cancer*.

